# Concomitant prediction of function and fold at the domain level with GO-based profiles

**DOI:** 10.1186/1471-2105-14-S3-S12

**Published:** 2013-02-28

**Authors:** Daniel Lopez, Florencio Pazos

**Affiliations:** 1 Computational Systems Biology Group, National Centre for Biotechnology (CNB-CSIC), C/ Darwin 3, 28049 Madrid, Spain

## Abstract

Predicting the function of newly sequenced proteins is crucial due to the pace at which these raw sequences are being obtained. Almost all resources for predicting protein function assign functional terms to whole chains, and do not distinguish which particular domain is responsible for the allocated function. This is not a limitation of the methodologies themselves but it is due to the fact that in the databases of functional annotations these methods use for transferring functional terms to new proteins, these annotations are done on a whole-chain basis. Nevertheless, domains are the basic evolutionary and often functional units of proteins. In many cases, the domains of a protein chain have distinct molecular functions, independent from each other. For that reason resources with functional annotations at the domain level, as well as methodologies for predicting function for individual domains adapted to these resources are required.

We present a methodology for predicting the molecular function of individual domains, based on a previously developed database of functional annotations at the domain level. The approach, which we show outperforms a standard method based on sequence searches in assigning function, concomitantly predicts the structural fold of the domains and can give hints on the functionally important residues associated to the predicted function.

## Background

Proteins are the key players of the cellular processes. Obtaining information on the structure, function and important residues for the protein repertory of a given organism (proteome) is crucial not only for getting insight into its biology, but also to foresee possible ways for modifying it in our benefit. Nevertheless, obtaining experimentally this kind of information is very slow and expensive. On the contrary, obtaining the raw sequences of complete proteomes or part of them is nowadays relatively fast and inexpensive, and this is getting even better with "next generation sequencing" technologies [1]. For these reasons, developing computational techniques for assigning structural and functional features to protein sequences is an active area of research.

Methods for predicting protein three-dimensional structure from sequence generally are based on the known relationship between sequence similarity and structural similarity. Most of these methods look for homologous proteins of known structure and model the problem sequence based on them. This search is either based on simple sequence matching methods for cases of close homology, or profile-based methods for remote homology.

Similarly, most methods for computationally assigning function to proteins ("annotation") are also based on the observed relationship between sequence similarity and functional similarity [2-4]. Functions of unknown proteins are inferred (transferred) from those of their homologs. This relationship between sequence similarity and functional similarity is far more complex than that between sequence and structure, in part due to the problem of precisely defining and quantifying "protein function" [5]. Contrary to what happens with protein structures, which can be univocally defined, quantified and compared, protein functions are more difficult to define. Many functional schemas and vocabularies co-existed in the past and still do, a lack of consensus which actually reflects this problem of lacking a precise definition of the concept "protein function". The de-facto standard nowadays for representing protein function is that generated and maintained the Gene Ontology (GO) consortium [6]. GO defines a set of functional terms (vocabulary) related by parenthood relationships. These relationships form a partially hierarchical structure which can be navigated from terms representing very general to those representing highly specific functional aspects of proteins. Additionally, GO terms can be divided in three classes created to represent three independent aspects of the complex phenomenon of protein function: 'molecular function', 'biological process', and 'cellular component'. A given protein is annotated by assigning to it one or more terms from these three sets. In the following, we will focus on the 'molecular function' aspect of proteins (GO:MF) since that is the one used in this work.

The basic concepts and methodologies for transferring function from homologous sequences have evolved with time and, at the same time, adapted to these new structured vocabularies (for recent reviews describing in detail the field see [7-10]). The evolution consisted mainly of incorporating more sensitive methods, based on profiles, and phylogenetic approaches for locating distant homologs from which to transfer function. Some methods also consider the GO:MF functional terms associated with all the homologs and their underlying hierarchical relationships to come up with a final set of terms for the problem sequence [11-13]. Another tendency is to concentrate on motifs or groups of residues, defined based on sequence and/or structural criteria, indicative of function, instead of relying on global sequence similarities spread through the whole length of the protein [14-17].

Most of these methods, specially those based on global sequence matches against individual proteins or profiles, are intended to assign function at the whole chain level, without distinguishing which individual domain is associated to a given GO:MF term. In most cases, this is not a problem of the methodologies themselves but of the annotations contained in the resources they search against. In these resources, functions are associated to whole chains, not to particular protein domains, and as such they are transferred to the problem sequences. Nevertheless, domains are the structural, evolutionary, and often functional units of proteins. In many cases, individual molecular functions can be assigned to them. Even the functional annotations in domain-oriented databases such as Pfam or Intepro suffer from this problem when these annotations are interpreted in terms of physical domains [18].

In this work, we present a method for annotating proteins with GO:MF terms at the domain level. The method is based on matching against a library of "position specific scoring matrix" (PSSM) profiles [19] derived from structural alignments of domains annotated with the same GO:MF functional term. These annotations are taken from the first resource specifically devoted to assigning GO:MF terms at the domain level, SCOP2GO [18]. Since all the domains within a profile share the same fold, the method also implicitly assigns fold to the domains of the query proteins, although that is not its main goal. Moreover, the pattern of positional conservation within these profiles can give clues on the functional sites of the query sequence. We show that a psi-blast [19] search against this library of profiles renders better results than an equivalent search against a database containing the original sequences of the domains, demonstrating the added value of constructing the profiles guided by the GO:MF functional annotations at the domain level. So, this method allows to concomitantly obtain information on function, fold and functional sites at the domain level for unknown proteins.

## Methods

**Figure 1:**
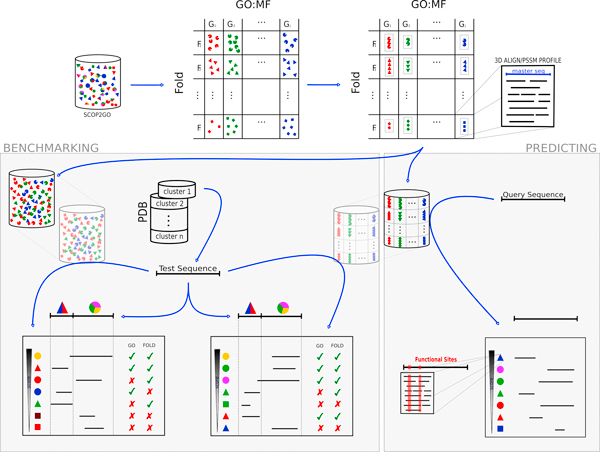
**Schema of the method**. The starting point is the SCOP2GO functional annotation of chains at the domain level (top left). The shapes represent the folds of the domains, while the colors represent assigned GO:MF functions. Structural alignments for the domains with the same fold and the same function are generated and PSSM profiles are derived from them. For benchmarking (left), an equivalent database is constructed merging all the domain sequences involved in these profiles. For assessing the performance of both resources for a given test sequence, new versions of both databases are built by excluding this sequence and all its homologs (transparent cylinders in the figure). Querying the test sequence against both resources produces list of hits which can be interpreted as predictions of folds and functions (colored shapes) associated to its domains. These predictions of both resources for the domains of the test sequence can be contrasted against its original SCOP2GO annotations (multi-colored triangle and circle). For predicting (right), a sequence of unknown domain characteristics in terms of fold and function is queried against the database of PSSMs. The hits can be interpreted as predictions of fold and function at the domain level. Additionally, the conservation pattern of the structural alignments associated to the matched PSSMs can give clues about functionally important residues.

Figure 1 illustrates the methodology used for building the library of profiles and searching against it, as well as the protocol used for benchmarking the method and comparing with psi-blast.

### Library of GO:MF profiles at the domain level

The idea is that each entry in this library represents an alignment of all domain sequences known to have a given GO:MF function (a non-redundant representation of them, actually) that can be related in an alignment, i.e. belonging to the same fold and hence amenable of structural alignment.

The starting point is the SCOP2GO resource, which contains GO:MF annotations at the structural domain level [18]. SCOP2GO uses an automatic method for discerning which particular domain of a protein chain is responsible for a GO:MF annotation originally assigned to the chain as a whole. Starting with the fold distribution of all the chains associated to a given GO:MF term, the method looks for the minimum set of structural folds necessary for explaining the (observed) fact that all these chains have that function. The GO:MF term is assigned to the domains with these folds. The process is iterated for the other GO:MF terms and the annotations accumulated in the domains [18].

Multidomain entries, as well as those corresponding to PDB chains annotated as "mutant" and "circular permutation" are excluded. The resulting domains can be seen as arranged in a matrix of Fold *X *Function (GO:MF) (Figure 1). Folds are structural folds as defined in SCOP [20]. Each entry in this matrix (i.e. set of structural domains with the same fold and the same GO:MF term) is made non-redundant at 40% identity with T-coffee [21]. Entries with fewer than 3 domains are discarded. The next step is to generate a multiple structural alignment with the resulting domains (Figure 1). Most programs for generating "real" multiple structural alignments are limited to a relatively small number of structures, which is exceeded in many cases in our dataset. For that reason, we used Dali_lite [22] to generate individual binary alignments of each domain against a "master", and generated a pseudo-multiple structural alignment by piling up these binary alignments. As the master domain, we choose that with the length closest to the mean length of all domains within the subset. The same procedure is repeated for each entry in the matrix. As explained above, each entry in the matrix represents a non-redundant subset of domains with the same structural fold and annotated in SCOP2GO with a specific MF:GO term. Finally, psi-blast PSSM profiles are generated for all these alignments. A PSSM ("position specific scoring matrix") is a representation of the aminoacid distributions of the positions of a multiple sequence alignment [19].

The current version of the library contains 338 entries covering 115 different GO:MF terms and 150 SCOP folds.

### Querying a sequence against the library

Since each entry in the library is associated to a fold and a GO:MF term, querying a whole-length sequence against the alignments/profiles within this library with "reverse psi-blast" (rpsblast) produces a list of hits each representing a concomitant prediction of fold and function for a particular segment (i.e. a domain) of the query sequence (Figure 1). Additionally, due to the way in which these structural alignments are generated, explained above, their conserved positions are expected to correspond to sites with some functional importance for that GO:MF function hosted in that fold, although positions conserved due to purely structural reason would also show up here. For this reason, inspecting the alignment of the query sequence against these conserved positions can give clues on its functional residues as well (Figure 1).

### Benchmarking

One of the added values of the method presented here is that the profiles are constructed "informed" by GO:MF annotations, instead of relying on the domain groupings that would result from sequence relationships alone (e.g. families and superfamilies) To evaluate the effect of this, we compared the results of searches against this database of pre-computed profiles, with those obtained by the same method (psi-blast) against an equivalent database with exactly the same domain sequences but not grouped according with GO:MF terms. In order to do that, all the domain sequences after the 40% ID filtering (just before performing the structural alignment) are mixed together in a large database which is formatted for psi-blast (Figure 1). Each sequence retains information on the Fold/GO:MF it comes from in order to later evaluate the results of querying against this database.

We constructed a test set for evaluating the performance of these two resources form the entire PDB clustered at 30% ID downloaded form the RCSB site [23]. The test set is constructed by taking one representative chain per cluster. The first sequence of the cluster having some domain annotated in SCOP2GO is taken. Note that, even if the two resources described above are based on domains, this test set is composed of whole-length chains, since that is the real-world scenario for applying the method presented here. The final test set contains 1017 chains. We have used the largest possible dataset taking into account the requirements of the sequences (known SCOP and SCOP2GO domain annotations) and the sequence redundancy filter.

For each chain in our test set, we carry out the following procedure. First we re-construct the two databases as described before but removing from the very beginning any domain corresponding to a chain within the same 30% ID PDB cluster as the test chain (Figure 1). This is to simulate a scenario in which predictions are going to be generated for sequences without clear homologs in the databases. In the case of the library of profiles, this obviously involves re-building the PSSM profiles which contain any of these chains homologous to the test chain without them. Then, the sequence of the test chain is queried against the two resources (single sequences and profiles) resulting in two lists of hits with their associated scores (e-values), each hit representing a Fold/GO:MF pair (Figure 1). Since the annotations of the domain(s) of the test sequence are known (in SCOP2GO), each hit can be labeled as "true" or "false" in terms of function and fold (Figure 1). The region of the test sequence aligning with a given hit is taken into account when deciding whether that hit is correct or not. I.e. a case in which the test sequence has the same fold/function as the hit but not in the aligned domain is not considered a match (Figure 1). This is done by "blasting" the region of the test sequence aligned with the hit against the sequences of all its domains, taken from ASTRAL [24], to confirm/discard that the alignment is in the correct domain.

So, for a given chain in the test set we obtain two sorted lists of hits, one for each method/resource, called "GO_PROFILE" and "PSI_BLAST" hereafter (Figure 1). Each hit can be labeled as correct or incorrect in terms of fold and function as explained before. In order to base the comparison on the same number of cases, only the top hit of each list (highest score) is evaluated. For that, a single list of "top hits" and their associated scores is generated for each method.

A ROC (receiver operator characteristics) analysis [25] is performed on these lists in order to evaluate the capacity of both resources to discriminate correct from incorrect hits. The ROC analysis generates a plot of "true positives rate" (TPR) against "false positives rate" (FPR) when varying the classification threshold (score of the method). A random method, without discriminative capacity, would produce a list with positives and negatives uniformly distributed through it that would render a diagonal, from [0,0] to [1,1], in the ROC plot. Curves above the diagonal represent methods with some discriminative power. This discriminative capacity is better as the curve gets closer to the top-left corner of the plot ([0,1]). So, a ROC curve is generated by cutting the sorted list of scores at different thresholds and plotting the resulting TPR's against the FPR's, calculated as

$\begin{array}{c} \mathrm{TPR}=\mathrm{Tp/}\left(\mathrm{Tp}+\mathrm{Fn}\right)=\mathrm{sensitivity}\\ \mathrm{FPR}=\mathrm{Fp/}\left(\mathrm{Fp}+\mathrm{Tn}\right)=1-\mathrm{specificity} \end{array}$

where Tp, Fn, Fp and Tn are the "true positives, "false negatives", "false positives" and "true negatives" resulting from a given threshold.

## Results

In the first part of this section we show the results of the large-scale evaluation of performances for GO_PROFILE and PSI_BLAST based on a test set of 1017 protein chains as explained in "Methods". In the second part, we show some examples of cases where one of the methods finds a right match while the other fails and vice versa, and where these failures are due to different reasons, to illustrate the advantage and drawbacks of this methodology as well as its complementarity with others. Another example allows to illustrate an additional advantage of this method: the possibility of obtaining a prediction of functionally important residues associated to the predicted GO:MF term.

### Large-scale evaluation

**Figure 2:**
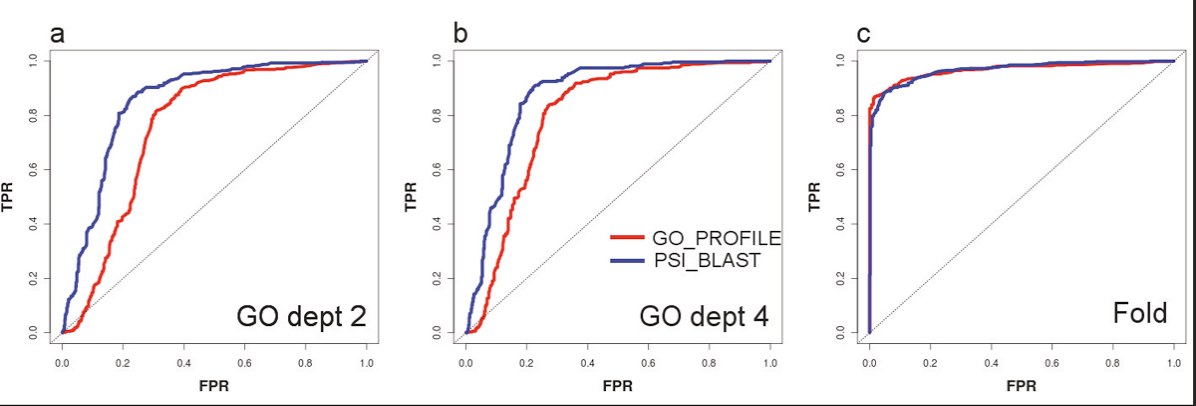
**Large-scale evaluation results**. ROC plots illustrating the discriminative capacity of the highest scoring hits detected by both methods in detecting the right function (a and b) and fold (c) in the correct domain. a) Only the GO terms at distance 2 or higher from the root of the GO:MF graph are evaluated; b) the same for distance 4 or higher (more specific terms).

Figure 2 shows the ROC plots generated for the lists of top hits of each method. Figures 2a and 2b show the performance of the methods in detecting the right GO:MF term, while Figure 2c shows the performance in detecting the right fold. The difference between Figures 2a and 2b is that in the last the evaluation is restricted to GO:MF terms far apart from the root of the hierarchy, i.e. those at distance 4 or higher from that root, in an attempt to evaluate only specific GO:MF terms. Although the distance to the root is not a perfect criteria to separate broad (e.g. "enzyme") from specific (e.g. "thymidylate synthase") GO:MF terms due to the uneven distribution of terms in the GO graph and the fact that it is not a perfect hierarchy, is a very convenient and easy way to have a first quantification of the level of broadness/specificity of a term. Actually very broad terms (distance to the root ≤ 2) are never used in this work since they are not included in the original SCOP2GO annotation of domains used for building the profiles [18]. From the original 115 different GO:MF terms contained in the library, 89 end up in the results used for generating Figure 2a, while 78 (more specific, distance > = 4) are used for generating Figure 2b.

It can be seen that both approaches present a very good discriminative power. Nevertheless, GO_PROFILE outperforms PSI_BLAST in assigning the right functions to the right domains (Figures 2a and 2b). When evaluating only more specific GO terms (Figure 2b) the difference in performance is lower and the results of a psi-blast search get closer to those obtained with the methodology presented here. In the Additional File 1 there are additional ROC plots for other levels of "functional specificity" (distances to the GO:MF root) which support this conclusion. This is probably due to the fact that, as we go to more specific functions, these are better reflected at the sequence level and hence they can be captured with standard sequence-based methods. On the contrary, proteins sharing a broad function (i.e. "hydrolase") might have been diverged largely at the sequence level or even lack a common evolutionary origin, and hence the landmarks they share in their sequences can only be captured with "supervised" profiles such as those presented here.

The ROC plots in the Additional File 1 include also the results of an hmmer search against HMM models [26] derived for the same alignments as the PSSMs. They are very similar and both are better than the psi-blast search against single sequences, highlighting the added value of the GO-based profiles which are able to capture subtle sequence landmarks of distant (or even evolutionary unrelated) proteins, as commented in the previous paragraph.

For the case of fold prediction, it can be seen that the performance of both approaches is very high and very similar (Figure 2c).

### Examples

The first example is the mitochondrial precursor of the ATP-synthase beta chain ([PDB:1w0k]D). The top hit of our method is the profile GO:0005524/c.37 (function "ATP binding" in fold "P-loop nucleoside phosphate hydrolases"), matched against the central domain of that protein. Nevertheless, an equivalent search with psi-blast finds as top hit a domain with function GO:0004156 ("dihydropteroate synthase activity", an enzymatic activity which is not even ATP-dependent) and fold c.1 ("TIM-barrel").

Another example is the periplasmic cytochrome C551I ([PDB:2mta]C). While GO_PROFILES correctly predicts GO:0020037 ("heme binding") in fold a.3 ("cytochrome C fold"), psi-blast's top hit is a DNA-binding protein (GO:0003677) with fold d.218.

The next example illustrates a problem of this method: the quality of the GO:MF domain annotations it relies on. For the Aspartyl-tRNA synthase [PDB:1b8a]B, the method matches its N-terminal domain with the profile GO:0005524/b.40 (function "ATP-binding" in fold "all-β/OB-fold". Such profile should not exist since there are not proteins with domains of that particular fold hosting that function. Nevertheless, there are examples of such domains wrongly annotated with that function in SCOP2GO. The reason for these wrong annotations is that these domains (responsible for anticodon binding in tRNA synthases) are frequently linked to the ATP-binding domains of these proteins, and there are many instances of them crystallized in isolation (as fragments) in PDB. The problem arises because these fragments are annotated with the function of the complete chain (ATP-binding) and consequently confound the methodology used in SCOP2GO (see [18] for details). On the contrary, psi-blast correctly matches this domain against the correct ATP-binding domain of a protein. This kind of errors due to problems in the SCOP2GO annotations would be alleviated as the SCOP2GO annotation is improved (e.g. by manual curation, etc) or future functional annotations at the domain level are used.

In the case of the mono-domain tyrosine phosphatase [PDB:1l8k]A, our method "correctly" matches it against the GO:0004725/c.45 profile ("protein tyrosine phosphatase activity" in fold "α/β phosphotyrosine phosphatases"). Nevertheless, this protein is annotated with a less specific term in GO (GO:0004721, "phosphoprotein phosphatase activity", the "ancestor" of GO:0004725). For this reason this counts as a failure in the automatic large-scale evaluation discussed in the previous point, even if our method is providing a more detailed (and correct) annotation. In this case, psi-blast matches against a protein with that less specific annotation (GO:0004721) and hence it counts as a true match.

**Figure 3:**
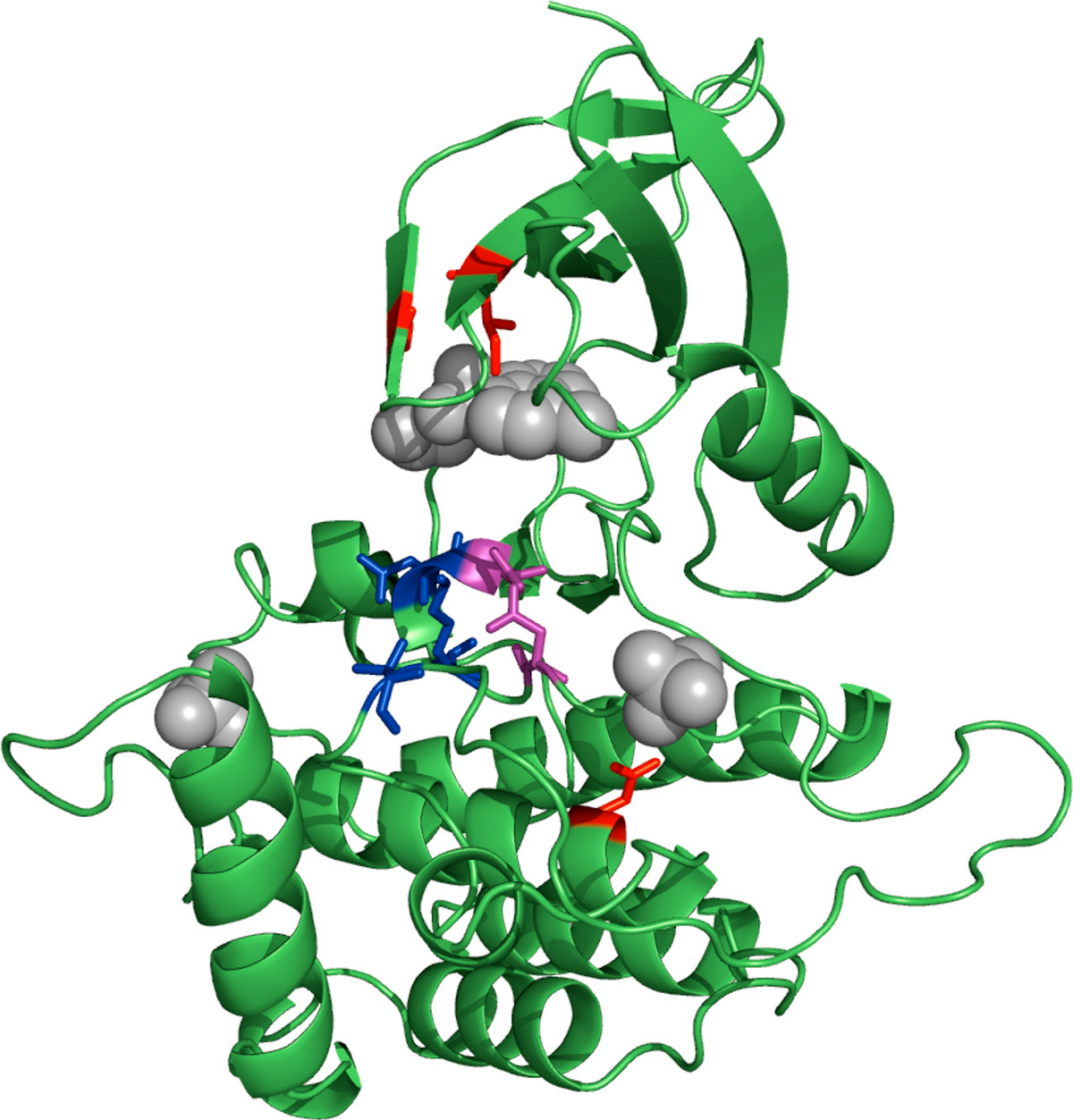
**Example of predicted functional residues**. Residues predicted by the method as associated to the GO:0004672 ("protein kinase activity") function for the casein kinase 1 ([PDB:2csn]A) mapped in the structure of this protein. Red and purple: predicted residues. Blue and purple: catalytic residues annotated in CSA. The prosthetic groups are shown in grey and spacefill. Figure generated with PyMOL (http://www.pymol.org).

The last example illustrates an additional advantage of this method: the fact that it can provide clues about possible functional sites, concomitantly with the prediction of fold and function. The casein kinase 1 ([PDB:2csn]A) is correctly matched against the GO:0004672/d.144 profile ("protein kinase activity" in fold "protein kinase like"). Figure 3 shows the positions conserved (95%) in this profile mapped on the 3D structure of this kinase, together with the residues annotated in the "catalytic site atlas" [27] for the same protein. It can be seen that all but one conserved residues either are annotated as catalytic, are very close to them, or are involved in binding cofactors (Figure 3).

## Discussion

It is well known that most proteins, especially in eukaryotic organisms, are multidomain [28]. In most cases, these domains perform distinct and quite independent molecular functions, to the extreme that some of these domains exist as independent proteins in other organisms (This is actually the basis of the "Rosetta Stone" method for predicting protein relationships [29].)

As commented in the Introduction, almost all methods and resources for predicting protein function are intended to work at the whole-chain level. Even the functional annotations of entries domain-oriented databases such as Pfam or Intepro are not intended to be interpreted in terms of physical domains. In [18] we show many examples of errors obtained when these resources and databases are used to infer annotations at the domain level. Obviously, this problem only applies to the "molecular function" aspect of the proteins, since the other two GO functional aspects ("cellular component" and "biological process") apply to complete chains and not domains.

The main methodological novelty of the procedure presented here is the usage of profiles derived from structural alignments of all domains associated to a given GO molecular function. This association of GO:MF terms to structural domains is taken from the first resource specifically devoted to this task [18]. Including all domains associated to a given function (those that can be structurally aligned, actually), and not only those with a common evolutionary origin, ensures that the molecular signatures within these profiles comprise the information of the whole sequence-space associated to a particular function (within a fold), and not only that restricted to a particular family or superfamily of proteins. This is actually one of the major differences, together with the GO:MF annotations at the domain level, with other resources intended to search against profiles derived for families or superfamilies [30]. In turn, these resources have the advantage that a match against their profiles provides additional information on family/superfamily membership and evolutionary origin. In this sense, all these resources complement each other in the evolutionary, structural and functional characterization of proteins at the domain level.

We compare this method with a base-line methodology for predicting protein function (psi-blast) in order to illustrate the added value of these novelties. Actually, only the added value of the GO-informed profiles, since the GO:MF annotations at the domain level are also provided to psi-blast in this benchmark. The large-scale evaluation based on GO annotations is not perfect due to many reasons (unspecific annotations, etc), some of them illustrated in the examples shown. Nevertheless, all these factors affect both methodologies due to the parallel evaluation procedure followed, based on the same dataset. An exhaustive comparison with more sophisticated methods for function prediction is outside the scope of this work due to the different functional vocabularies and databases used and, more importantly, the domain orientation of this method: almost all other existing resources for function prediction assign function at the whole-chain level. We also show some examples to illustrate the advantages of this method in particular situations and highlight its complementariness with existing approaches. Indeed, the method presented here is not intended to compete with the plethora of methods designed for predicting function at the whole chain level, but to fill a very particular niche: function prediction at the domain level. Nevertheless, this method can be also used to infer molecular functions for whole chains in two ways: i) although excluded from the benchmark presented here for simplicity, the SCOP2GO resource also contain functional assignments for groups of domains, and ii) the function of the whole chain can be manually inferred from the annotations of the individual domains although this requires some expert knowledge.

The domain orientation of this methodology also makes that it can be only applied to the "molecular function" category of GO (GO:MF) and not to the "biological process" category (GO:BP). As commented above, only the molecular functions can be differentially assigned to particular domains, while biological processes are properties of whole chains.

This resource will be improved as the GO:MF annotations at the domain level it is based on, which right now are generated with an automatic procedure, are extended and manually curated. Moreover, the method presented here can be implemented with any other domain-based functional annotation.

## Conclusions

We present here a method and a resource for the concomitant prediction of fold and molecular function at the domain level, using a single sequence as input. The method outperforms standard sequence-based methods. Functionally important sites may also be identified although this feature has not been exhaustively benchmarked so far and we only show illustrative examples.

## Competing interests

The authors declare that they have no competing interests.

## Authors' contributions

FP conceived the original idea. FP and DL designed the experiments. DL implemented and performed all the experiments. FP and DL contributed to the writing of the manuscript.

## Supplementary Material

Additional file 1 **Additional results of the large-scale evaluation**. Additional ROC plots for other levels of functional specificity (distance to the root of the GO:MF graph) including also the results of HMM searches against the profiles. Click here for file

## Declarations

This article has been published as part of *BMC Bioinformatics *Volume 14 Supplement 3, 2013: Proceedings of Automated Function Prediction SIG 2011 featuring the CAFA Challenge: Critical Assessment of Function Annotations. The full contents of the supplement are available online at http://www.biomedcentral.com/bmcbioinformatics/supplements/14/S3.
