# Supplementary material for: Concomitant prediction of function and fold at the domain level with GO-based profiles
Source: BMC Bioinformatics. 2013 Feb 28;14(Suppl 3):S12. doi: 10.1186/1471-2105-14-S3-S12 (PMC3584904; doi:10.1186/1471-2105-14-S3-S12)
Supplement: Additional file 1 — Additional results of the large-scale evaluation. Additional ROC plots for other levels of functional specificity (distance to the root of the GO:MF graph) including also the results of HMM searches against the profiles. [file 1471-2105-14-S3-S12-S1.pdf]

Supplementary Material for

Concomitant prediction of function and fold at the domain level with GO-based profiles

Daniel Lopez[1] and Florencio Pazos[1,*]

[1]Computational Systems Biology Group, National Centre for Biotechnology (CNB-CSIC), C/ Darwin 3, 28049 Madrid, Spain

* pazos@cnb.csic.es

Additional ROC plots for other levels of functional specificity (distance to the root of the GO:MF graph). The plots are sorted from lower (level 2) to higher (level 6) specificity. The plots also include the results of a *hmmer* search against HMM models derived from the profiles. Blue: GO_PROFILES Red: PSI_BLAST Green: HMM

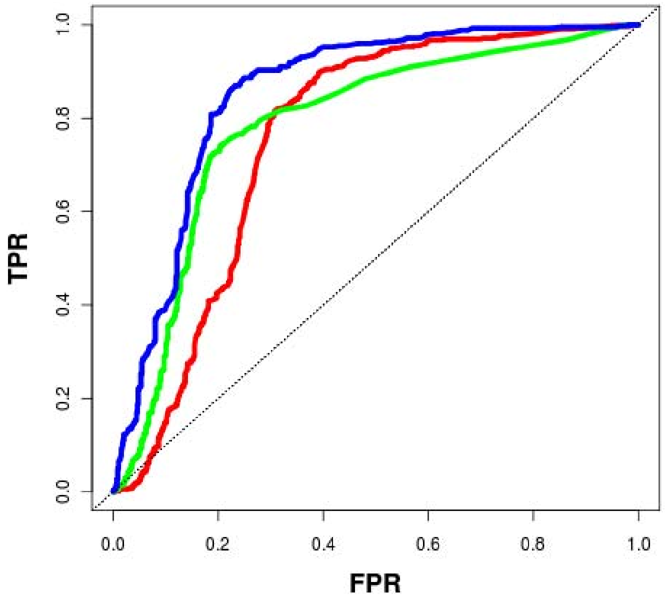

LEVEL 2

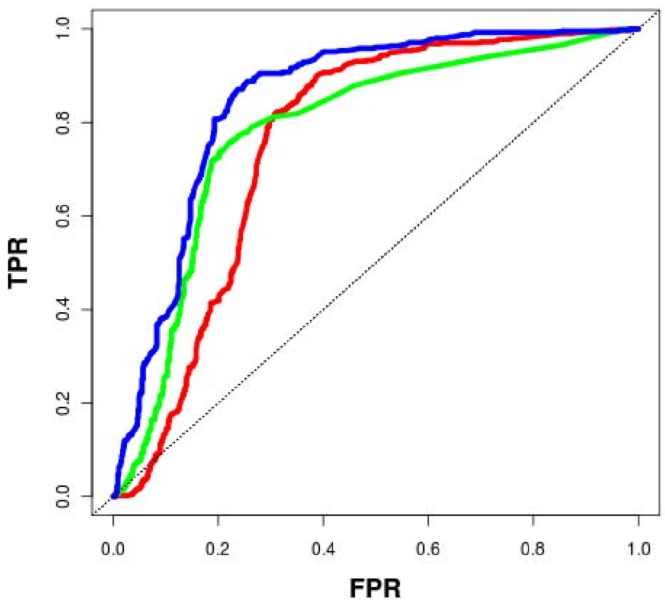

LEVEL 3

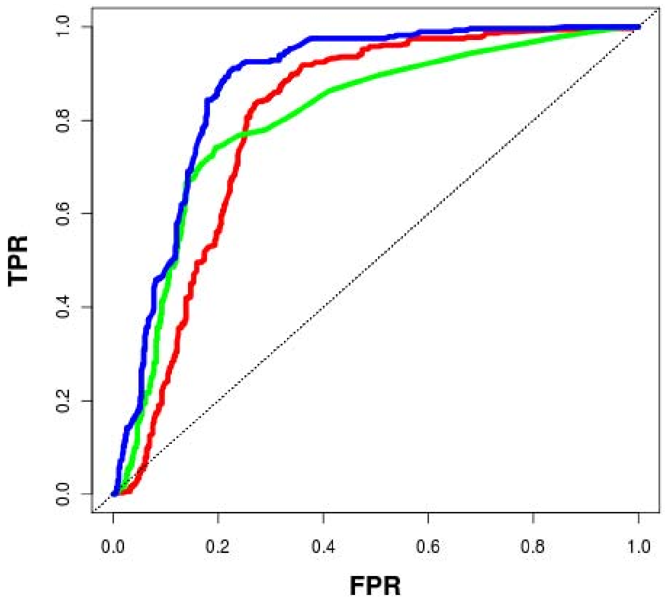

LEVEL 4

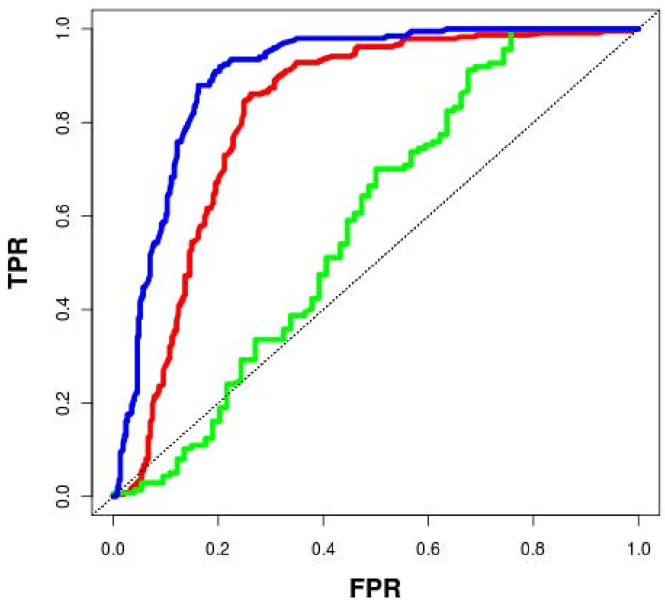

LEVEL 5

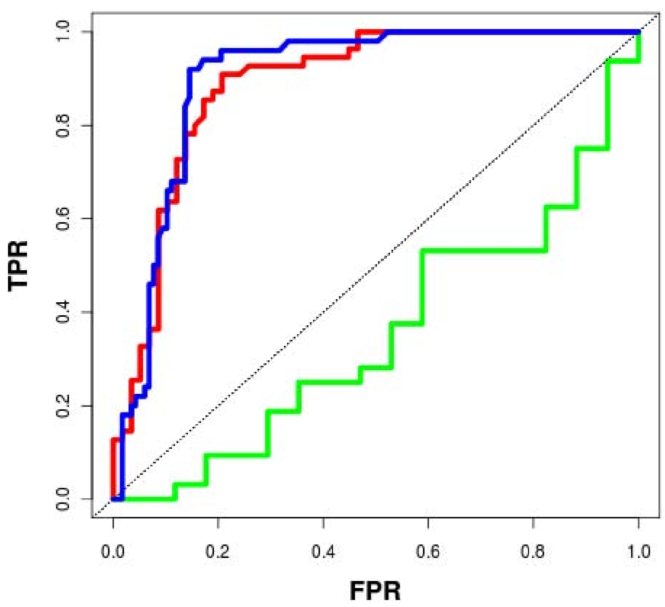

LEVEL 6
